# Arrhythmic Effects Evaluated on *Caenorhabditis elegans*: The Case of Polypyrrole Nanoparticles

**DOI:** 10.1021/acsnano.3c05245

**Published:** 2023-08-25

**Authors:** Sumithra Yasaswini Srinivasan, Pilar Alvarez Illera, Dmytro Kukhtar, Núria Benseny-Cases, Julián Cerón, Javier Álvarez, Rosalba I. Fonteriz, Mayte Montero, Anna Laromaine

**Affiliations:** † Universitat de Autonoma de Barcelona, Institut de Ciència de Materials de Barcelona (ICMAB), 08193, Bellaterra, Barcelona, Spain; ‡ Universidad de Valladolid, Instituto de Biomedicina y Genética Molecular (IBGM), 47005, Valladolid, Spain; § Modeling Human Diseases in *C. elegans* Group - Genes, Disease and Therapy Program, Bellvitge Biomedical Research Institute - IDIBELL, 08908 L’Hospitalet de Llobregat, Barcelona, Spain; ∥ ALBA Synchrotron, 08290, Cerdanyola del Vallès, Barcelona, Spain

**Keywords:** Polypyrrole nanoparticles, *C. elegans*, small animal models, arrhythmia models, cardiotoxicity

## Abstract

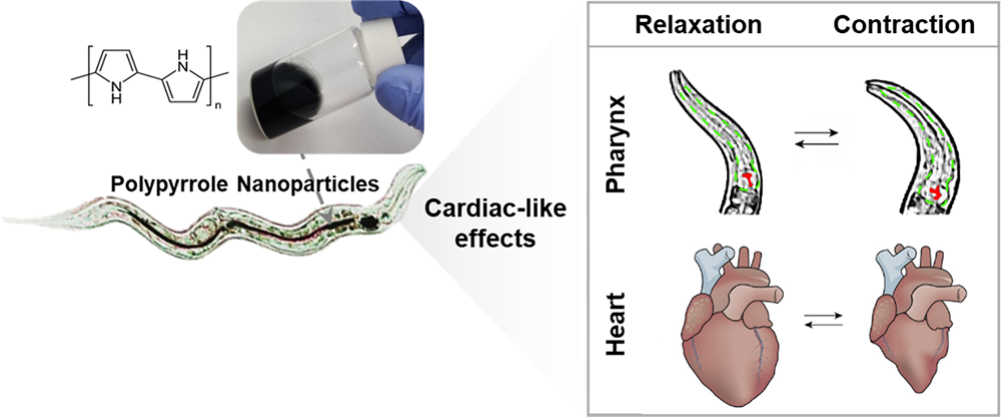

Experimental studies and clinical trials of nanoparticles for treating diseases are increasing continuously. However, the reach to the market does not correlate with these efforts due to the enormous cost, several years of development, and off-target effects like cardiotoxicity. Multicellular organisms such as the *Caenorhabditis elegans* (*C. elegans*) can bridge the gap between *in vitro* and vertebrate testing as they can provide extensive information on systemic toxicity and specific harmful effects through facile experimentation following 3R EU directives on animal use. Since the nematodes’ pharynx shares similarities with the human heart, we assessed the general and pharyngeal effects of drugs and polypyrrole nanoparticles (Ppy NPs) using *C. elegans*. The evaluation of FDA-approved drugs, such as Propranolol and Racepinephrine reproduced the arrhythmic behavior reported in humans and supported the use of this small animal model. Consequently, Ppy NPs were evaluated due to their research interest in cardiac arrhythmia treatments. The NPs’ biocompatibility was confirmed by assessing survival, growth and development, reproduction, and transgenerational toxicity in *C. elegans*. Interestingly, the NPs increased the pharyngeal pumping rate of *C. elegans* in two slow-pumping mutant strains, JD21 and DA464. Moreover, the NPs increased the pumping rate over time, which sustained up to a day post-excretion. By measuring pharyngeal calcium levels, we found that the impact of Ppy NPs on the pumping rate could be mediated through calcium signaling. Thus, evaluating arrhythmic effects in *C. elegans* offers a simple system to test drugs and nanoparticles, as elucidated through Ppy NPs.

## Introduction

The bench-to-market process for drug development is arduous, taking up to 15 years^1^ and incurring an enormous cost (∼$1–2 billion).^2^ In addition, progress in the early stages is difficult, as only ∼14% of drugs are approved from phase I trials to enter phase II trials.^3^ Even though drug development research is experiencing tremendous advances with modern therapeutic strategies like nanomedicine, shortcomings such as postmarket drug withdrawals due to adverse side effects are critical and must be addressed.

Cardiac effects, such as cardiotoxicity and arrhythmogenicity, are among the most common side effects^3^ causing 47% of drug withdrawals from the market.^4^ Even existing treatments for arrhythmia are known to cause pro-arrhythmic side effects.^5^ Nanomedicine offers a palette of drugs, nanoparticles, and strategies; a rigorous investigation of their efficacy and side effects can accelerate the progress of safe and innovative therapies to clinical trials. Among other nanomaterials, polypyrrole nanoparticles (Ppy NPs) are scrutinized as an emerging candidate for cardiac arrhythmia treatments^6−9^ and other biomedical applications.^10−12^

Polypyrrole (Ppy) is an intrinsically conducting polymer with good aqueous stability and allows surface functionalization.^12,13^ Reports confirm that Ppy NPs increase cardiac conduction velocity, synchronize irregular cardiac rhythm, and enhance Ca^2+^ signaling across cardiac cells, although *in vitro.*(7,14) However, some studies indicated that high concentrations of Ppy NPs could affect its biocompatibility and cause undesirable effects.^15−17^ A recent study of *p*-toluene sulfonic acid-doped Ppy NPs reported that at high concentrations (500 μg/mL) caused embryonic toxicity, premature hatching, and a reduced heart rate in zebrafish larvae.^18^ Although Ppy NPs are reported as biocompatible in other works,^10,16,19^ a thorough *in vivo* assessment of biocompatibility and cardiac effects of Ppy NPs is vital to solve discrepancies and move forward.

*In vivo* screening in large animals is the optimal solution to reach clinical trials; however, it is expensive and time-consuming, has low throughput, and raises ethical concerns.^20^ Thus, in order to advance at the early stages of the nanomaterial’s development, it is imperative to use biologically relevant models which fulfill the 3R (Replace, Reduce, and Refine) requirements and facilitate safe-by-design therapeutic agents, bringing out drugs with minimal side effects into the market.^21^

To date, alternative models such as zebrafish and *Drosophila melanogaster* have been explored. However, those small animals also exhibit disadvantages that limit their use. In zebrafish, the Ca^2+^ mechanism and transient levels differ from humans, displaying a low expression of ryanodine receptors and a decreased sensitivity to cytoplasmic calcium concentration in the zebrafish heart compared to humans.^22^ The zebrafish casq2 and ryr2 genes were identified as orthologous to human CASQ2 and RYR2; however, mutations in this animal are not reported to exhibit CPVT phenotypes.^23,24^ Additionally, experimentally quantifying the ingested concentration of drugs or nanomaterials in zebrafish is challenging, limiting its use in pharmacokinetics and pharmacodynamics (PK/PD) studies.^4,25^

The heart of another small animal model, *Drosophila melanogaster* (fruit fly), comprises two pacemaker cells with an open circulatory system, leading to heartbeats in both directions, anterograde and retrograde, which differs from human hearts.^26^ Experimentally drosophila becomes less transparent with aging, complicating the measurement of the heart rate and rhythm.^27^ In general, not all channelopathy mutations of arrhythmia have been reported to display the expected phenotypes on drosophila or zebrafish.

We advocate for the small animal *Caenorhabditis elegans* (*C. elegans*), a 1-mm-long nematode with a high genetic homology to humans, short life span, and fast reproduction cycle, which enable rapid evaluation through the entire life cycle and the use of a large sample size.^28^ Additionally, *C. elegans* strains can be maintained as frozen stock, allowing storage of relevant mutants, which is difficult or impossible in other species.

**Figure 1:**
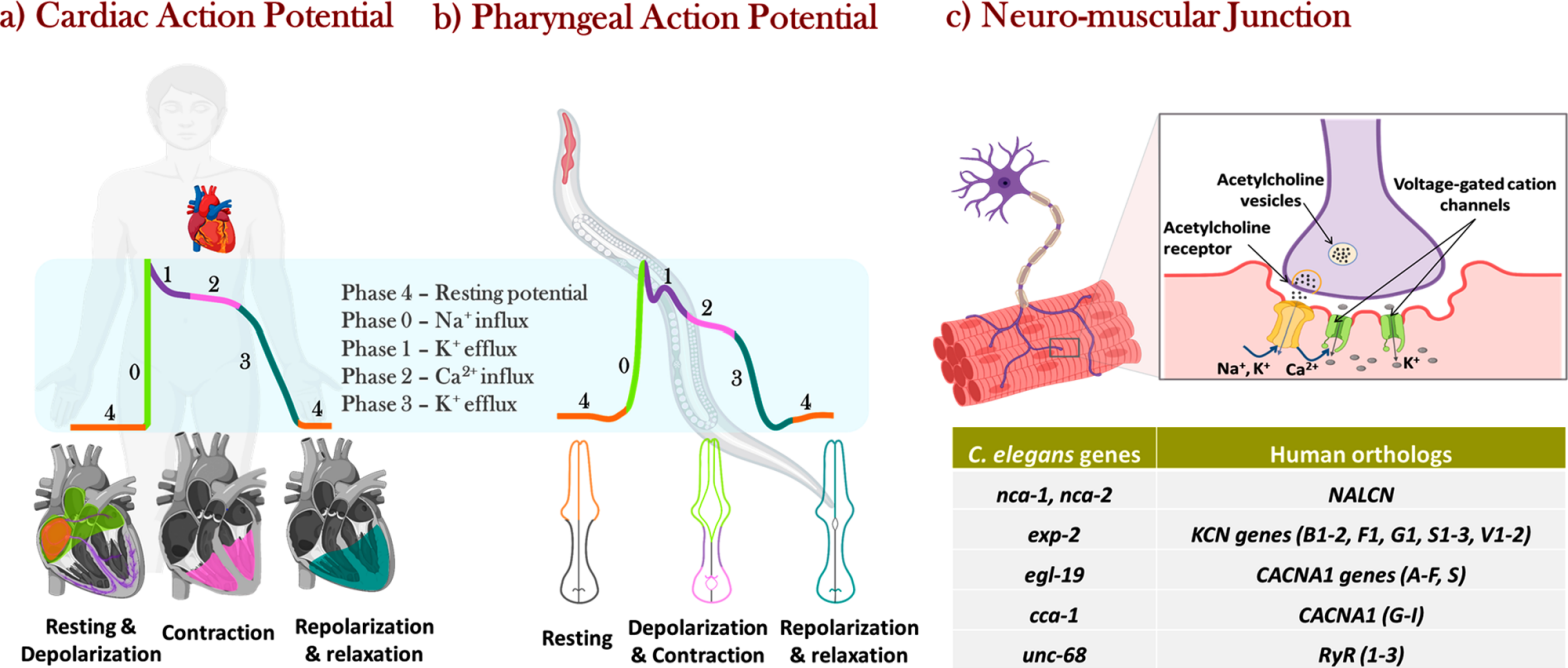
(a) Schematics of the cardiac contraction and action potential generation and propagation in humans. (b) *C. elegans* pharyngeal contraction and action potential graph showing a strong resemblance in ion transfer events as the human. (c) Scheme of the neuromuscular junction similar in humans and in *C. elegans*. The table reports the orthologous genes encoding cation channels and gap junctions at the neuromuscular junctions of the *C. elegans* pharynx and the human heart. Created with BioRender.com.

The pharynx of *C. elegans* is a continuously pumping feeding organ responsible for the movement of ingested substances toward its intestine through peristalsis. Although *C. elegans* do not possess a heart or vasculature for blood circulation, their pharynx and human heart share structural and molecular similarities; both are made of muscle cells connected to neuronal cells via gap junctions (Figure 1). The cell membranes are composed of voltage-gated cation channels, leading to a series of cation transfer events, such as Na^+^, K^+^, and Ca^2+^, generating action potentials and causing muscle contraction (Figure 1a,b).^29^ This muscle contraction occurs due to repetitive Ca^2+^ transients tightly regulated through voltage-gated calcium channels, which are orthologous in humans and *C. elegans*(30) (Figure 1c). The *unc-68* gene of *C. elegans* encodes for calcium ion binding receptors, orthologous to human ryanodine receptors, RYR1, RYR2, and RYR3.^30^ Likewise, the calsequestrin receptor encoding gene csq-1 in *C. elegans* is orthologous to human CASQ1 and CASQ.^30^ Mutations in both *unc-68* and *csq-1* genes lead to CPVT phenotypes as observed in humans.^31^ Therefore, the *C. elegans*’ pharynx can provide extensive information for cardiac-like effects, such as the pumping rate and Ca^2+^ transient levels, which could allow biologically relevant mutants useful for the initial screening of drugs and nanomaterials.^31−33^ Additionally, worms are entirely transparent even in the adult stage; the pharyngeal pumping rate can be measured through visualization by optical microscopy.^34^

In light of the genetic and molecular similarities, we used *C. elegans* to evaluate the pharyngeal effects of two commercial substances: Propranolol (PL) and Racepinephrine (RE). Subsequently, we assessed the promising Ppy NPs in *C. elegans* for their systemic effects, such as survival rate, growth and development, oxidative effects, and reproductive and intergenerational toxicity. Pharyngeal effects of Ppy NPs, the influence of treatment duration, and excretion were also evaluated, and molecular effects, such as lipid and intracellular pharyngeal calcium levels, were investigated to understand the mechanistic action of Ppy NPs.

## Results and Discussion

### Physico-Chemical Properties of Ppy NPs

PPy NPs were synthesized by chemical oxidative polymerization method using pyrrole monomer (0.1 M), FeCl_3_·6H_2_O as the dopant-*cum*-oxidant at a 24:1 ratio (oxidant:monomer), and PVA as a surfactant (7.5 wt % of monomer) (Figure S1).^35^ The NPs were spherical with a mean size of 132 ± 31 nm from transmission electron microscopy (TEM) images, a hydrodynamic mean diameter of 196.4 nm, and a polydispersity index (PDI) of 0.17 by dynamic light scattering (DLS) (Figure S2). Fourier transform infrared (FT-IR) spectra and absorbance spectra of Ppy NPs in the UV–vis–near-infrared (UV–vis-NIR) region elucidated the chemical structure, composition, and optical properties of Ppy NPs, respectively (Figure S2). Extensive characterization is presented in the Supporting Information.

### Stability of Ppy NPs

**Figure 2:**
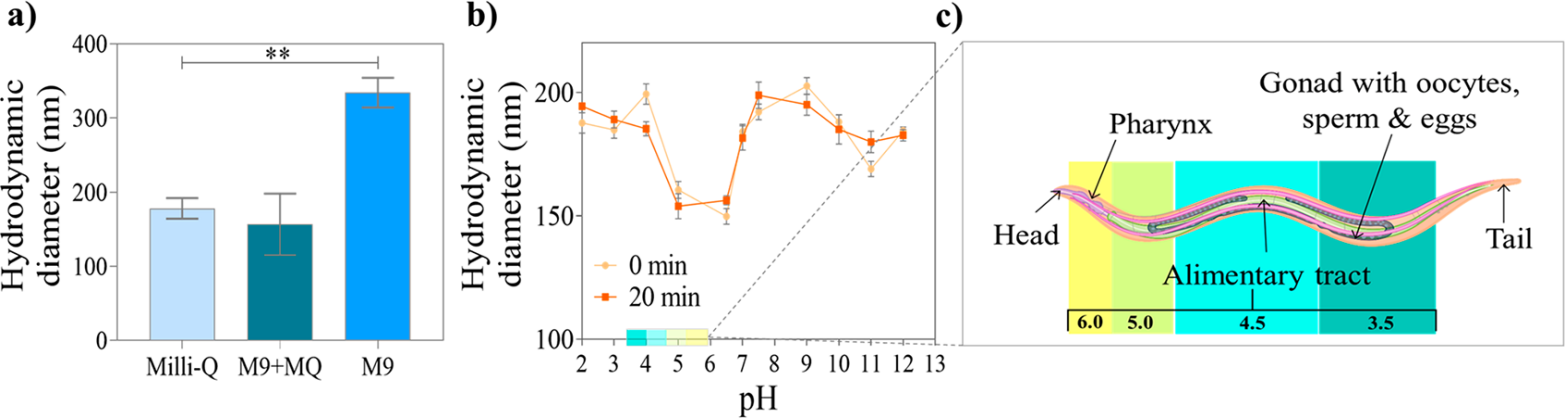
Stability of Ppy NPs (a) in Milli-Q (MQ) water, 50:50 mix of M9:MQ, and M9 buffer. (b) Stability of Ppy NPs at different pH values over time. (c) Schematic representation of the pH of the intestinal tract of worms, which is within the same range as humans but in the opposite direction.

Aqueous solutions of Ppy NPs were stable in water for up to 6 months of usage, showing no visible precipitation. The M9 buffer, a standard buffer used for *C. elegans* exposure, reduced the colloidal stability of Ppy NPs since the hydrodynamic diameter indicated some aggregation (Figure 2a). Therefore, 50% dilution of M9 with MQ water was tested (Figure 2a) and used in exposure experiments, which ensured a good dispersion of Ppy NPs and a healthy environment for *C. elegans.*

The intestine of *C. elegans* exhibits a wide range of pH values from 6 (anterior) to 3 (posterior) (Figure 2c), similar to humans. Therefore, we measured the stability of Ppy NPs in the pH range 2–13 by DLS using acidic/basic solutions at 0 min and after 20 min since the average residence time within the worm’s intestine is less than 10 min.^36−38^ Ppy NPs’ size remained constant at all pH values during this period (Figure 2b), suggesting desirable behavior during exposure to *C. elegans*.

### The Fate of Ingested Ppy NPs

**Figure 3:**
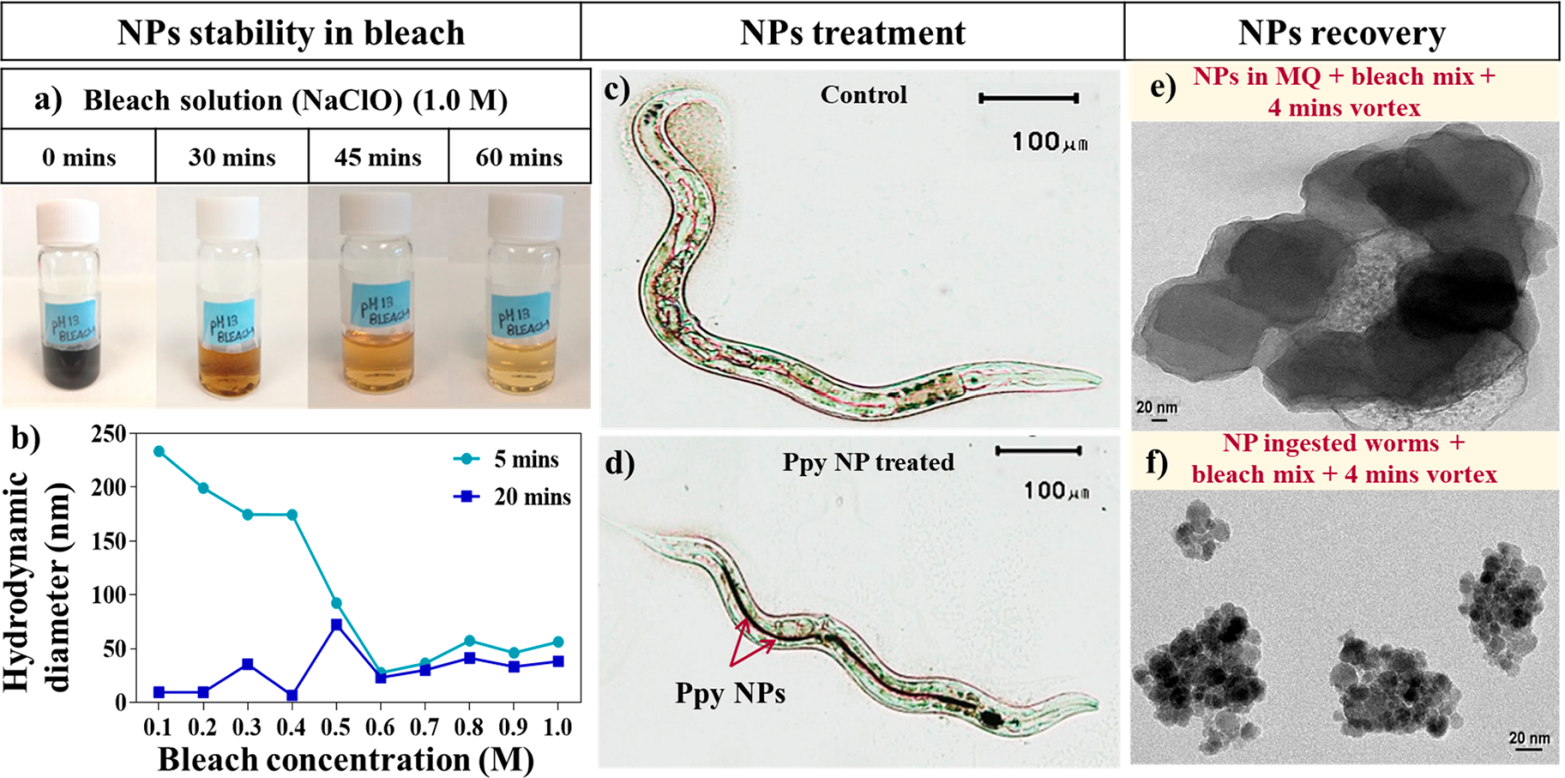
(a) Ppy NPs dispersed in a bleach solution over time (top). (b) Hydrodynamic diameter of Ppy NPs in different concentrations of bleach solution. Optical microscopic images of the (c) untreated *C. elegans* (control), (d) *C. elegans* treated with Ppy NPs. TEM images of the (e) NPs after treatment at 0.3 M bleach + 5.0 M NaOH and 4 min vortex. (f) NPs recovered after bleach treatment of the treated worms.

*C. elegans* were exposed to 100 μg/mL Ppy Nps for 24 h and visualized within the intestines through optical microscopy (Figure 3c,d), confirming the ingestion of the NPs by the worms. *C. elegans* provides the opportunity to recover and investigate the fate of the ingested NPs in the intestinal environment after exposure. In order to recover the NPs, a mixture containing sodium hypochlorite (NaClO) (commercial bleach) and sodium hydroxide (NaOH) is used.

The evaluation of NPs in a concentrated bleach solution (1.0 M) showed evident dissolution over time as the black color of Ppy NPs disappeared, leaving the clear bleach solution after 1 h (Figure 3a). Hence, the stability of Ppy NPs was assessed by DLS in a range of bleach concentrations (0.1–1.0 M) at 5 and 20 min, and we found that the NPs were stable at concentrations lower than 0.4 M for 5 min (Figure 3b). Since the NPs dissolved above this concentration and time, 0.3 M was chosen as our working concentration. TEM images revealed that the NPs displayed 128 ± 10 nm diameter after mixing them with NaClO (0.3 M) and NaOH (5 M) and vortexed for 4 min (Figure 3e), confirming the stability of the NPs in the recovery procedure of the worms.

Then, the treated worms (Figure 3d) were dissolved using the same mixture and vortexing. The recovered NPs were visualized by optical microscopy, ensuring that the worms were dissolved (Figure S3). TEM images of the recovered NPs indicated some aggregation with a reduced diameter of individual particles (12 ± 3 nm), suggesting some dissolution and aggregation in the alimentary system (Figure 3f). The aggregation of the excreted NPs was also confirmed by DLS analysis, exhibiting a hydrodynamic diameter of 311 ± 75 nm and PDI of 0.3 (Figure S3).

### Effects of Propranolol and Racepinephrine in *C. elegans*

**Figure 4:**
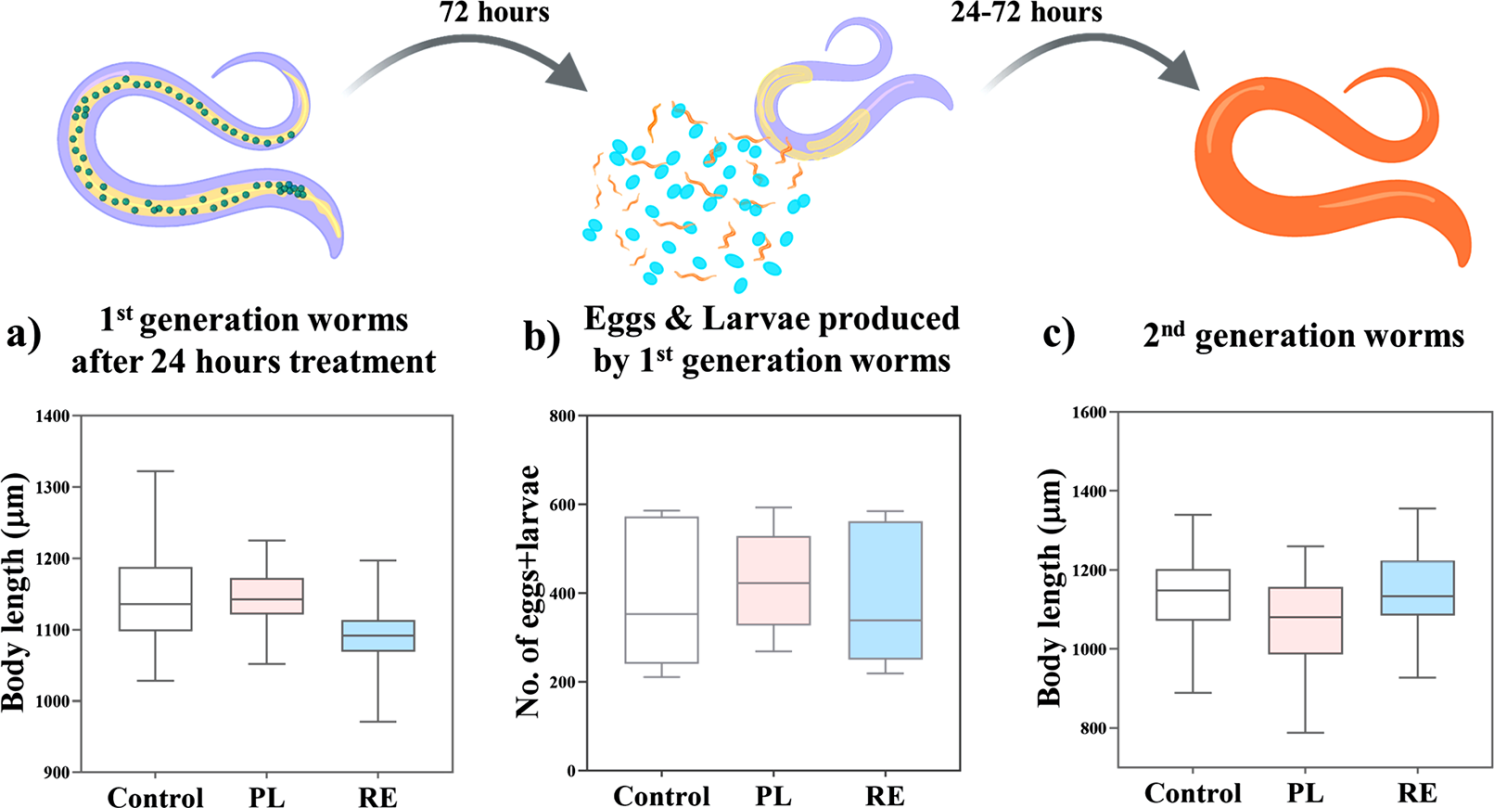
Systemic effects of PL and RE on *C. elegans*. (a) Body length of first generation worms after 24 h treatment (*N* = 40). (b) Number of eggs and larvae produced by the worms in 72 h postexposure (*N* = 12). (c) Body length of fully developed second generation worms (*N* = 30). No statistical significance was found in any case. Top panel (scheme) created with BioRender.com.

Prior to the evaluation of the Ppy NPs in *C. elegans*, we assessed two commercial substances, Propranolol (PL) and Racepinephrine (RE). PL is a β-adrenergic receptor antagonist with serotonin receptors as targets,^39−41^ which reduces the cardiac pumping rate, and RE is a β-adrenergic receptor agonist that increases the pumping rate.^42,43^ The worms were exposed to PL or RE at 100 μM concentration,^4,44^ at L4 larval stage for 24 h. The survival rate (Figure S4) and development of the worms from the L4 to the egg-bearing adult stage were not affected upon treatment (Figure 4a). Reproductive toxicity was determined by computing the number of eggs and larvae produced in 72 h. The treatments did not affect the reproductive rate, yielding a similar number of offspring to untreated worms (∼390) (Figure 4b), nor the body length of the second-generation adult worms (Figure 4c), indicating that PL and RE do not cause any adverse effects in *C. elegans* during this time course.

**Figure 5:**
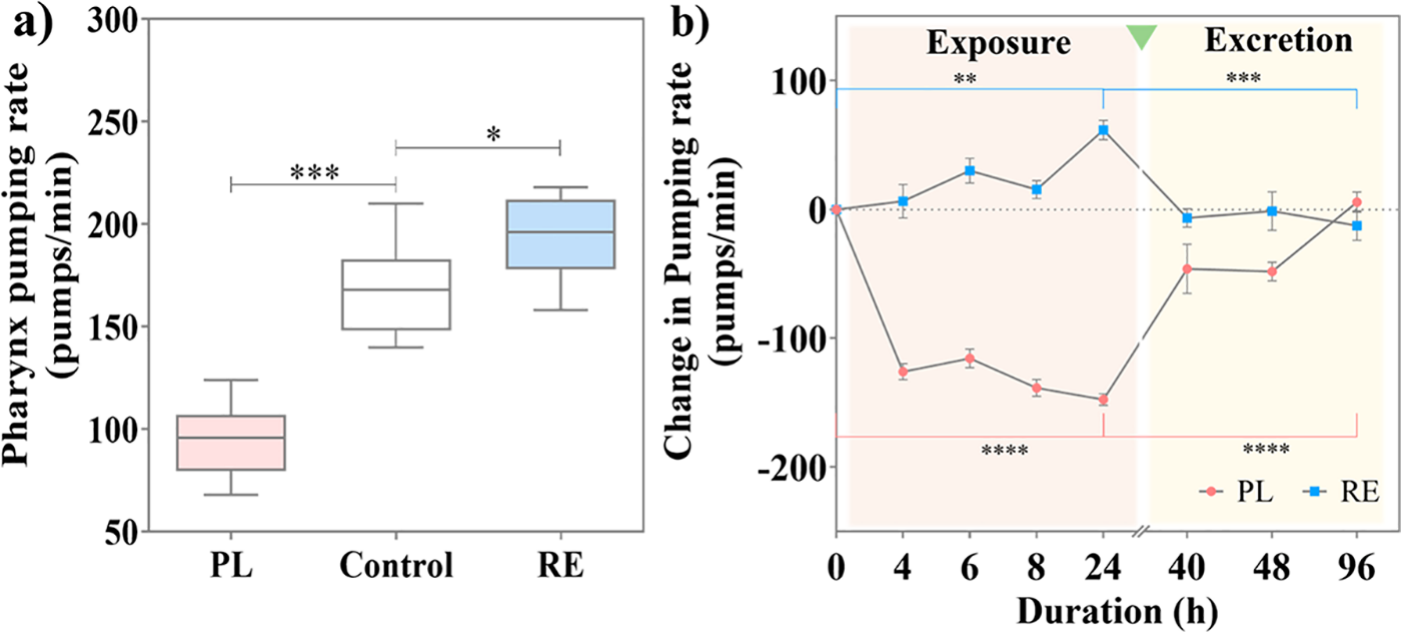
(a) Pharynx pumping rate upon 24 h treatment with PL or RE at 100 μM concentration. (b) Impact of treatment and excretion duration of PL and RE in the change in pharynx pumping rate.

The pharynx pumping rate of *C. elegans* after exposure to PL and RE (Figure 5a) revealed that PL decreased the pharyngeal pumping rate to an average of 94 pumps/min; RE treatment increased the rate to 193 pumps/min compared with the untreated worms (168 pumps/min), correlating well with the cardiac effects observed in humans in both cases. Exploiting the experimental simplicity and robustness of *C. elegans*, we computed the change in pumping rate along the treatment duration and postexcretion of PL and RE (Figure 5b). The pharynx pumping rate was measured at 4, 6, 8, and 24 h of exposure. The effects of PL and RE were time-dependent, with the maximum change in pumping rate observed at 24 h. The treatments were stopped by transferring the worms to NGM plates containing only bacterial food. During exclusive bacterial feeding, the worms excreted the ingested substance, and the pumping rate was measured at 16, 24, and 72 h after excretion, i.e., 40, 48, and 96 h from the beginning of the exposure. The change in the pharynx pumping rate was computed as the difference between the pumping rate of treated worms and the mean pumping rate of untreated worms. The change in pumping rate induced by the drugs recovered to the initial rate after 72 h for PL-treated worms, whereas for RE-treated worms, it was attained after 16 h of excretion, suggesting the effect caused by RE is short-lived compared to PL.

The *C. elegans* response to commercially available cardiac drugs, PL and RE, was as expected in efficacy and toxicity profiles, thus encouraging further evaluation of the effects of Ppy NPs.

### Systemic Effects of Ppy NPs in *C. elegans*

**Figure 6:**
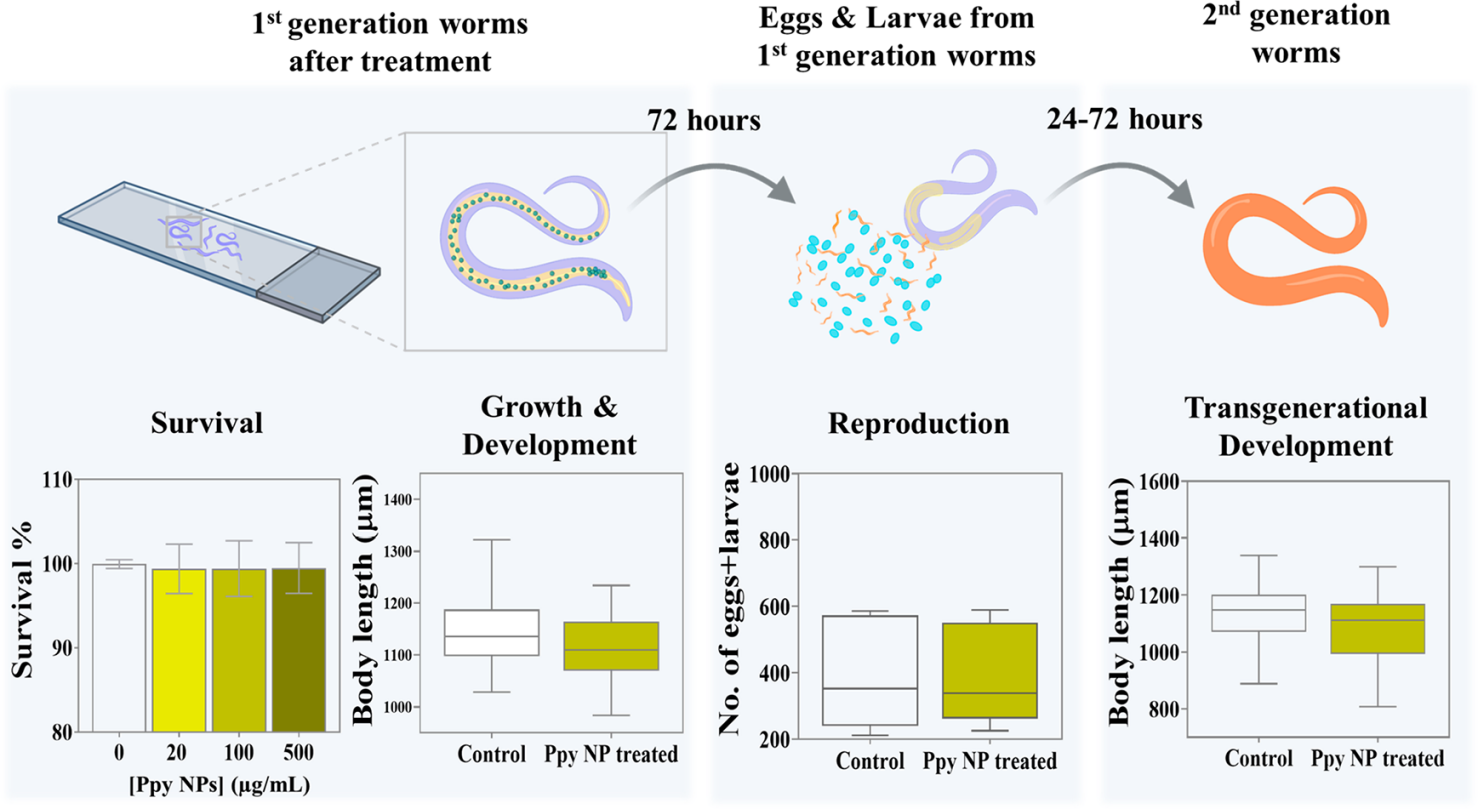
Systemic effects of Ppy NPs on *C. elegans*. (a) The percentage of survival of untreated worms and various concentrations of Ppy NPs treated worms (*N* = 60). (b) The measurement of the body length of 1st generation worms after 24 h of treatment (*N* = 40). (c) The number of eggs and larvae produced by the worms in 72 h post-exposure (*N* = 12). (d) The measurement of the body length of fully developed second generation worms (*N* = 30). No statistical significance was found in any case. Top panel (scheme) created with BioRender.com.

We measured the survival rate of the worms after treatment with Ppy NPs (20, 100, 500 μg/mL) for 24 h, showing no adverse toxicity at any concentration (Figure 6a). All the subsequent experiments were conducted with the intermediate concentration, 100 μg/mL Ppy NPs, to ensure an effective and viable dosage. After Ppy NPs exposure, worms reached their fully grown stage with an average body length of ∼1100 μm (Figure 6b), showing that the growth and development of the worms were not affected by the NPs.

*C. elegans* offers the possibility to evaluate the effects of NPs on the reproductive system of worms. The number of larvae and eggs produced by each worm in 72 h was computed. Similar to survival, growth, or development, NP treatment did not affect reproductive ability, even at high concentration (Figure 6c). Additionally, we examined if exposure to Ppy NPs could impart any intergenerational toxicity to the worms. The second-generation worms exhibited identical development (body lengths) after 72 h from hatching (Figure 6d), indicating that exposure to NPs does not affect the growth of the following generation.

### Pharynx Pumping Rate

**Figure 7:**
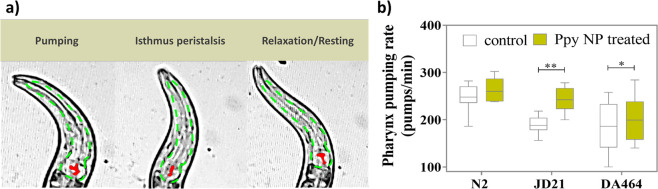
(a) Video snapshots of pharyngeal muscle contractions in *C. elegans*: pumping, isthmus peristalsis, and resting. The grinder is marked in red in the images, displaying contraction and relaxation, and the whole pharynx is localized with a green dotted line. (b) The pharynx pumping rate in *C. elegans* of N2, JD21, and DA464 strains, with and without Ppy NPs treatment (*N* ≈ 30).

Treating the wild-type *C. elegans* (N2) with Ppy NPs for 24 h (100 μg/mL) increased the pharyngeal pumping rate from 248 ± 28 pumps/min to 264 ± 24 pumps/min (Figure 7b), similar to the effect observed with RE. In order to understand the possible molecular mechanism of action of Ppy NPs, we employed two mutant strains with slow pumping rates. The JD21 strain has a deletion mutation in *cca-1*, a voltage-gated calcium channel homologous to human low-voltage T-type calcium channels *CACNA1G*, *CACNA1H*, and *CACNA1I* (Cav3 genes),^24^ with mutations implicated in long QT syndrome (LQT9).^45,46^ On the other hand, the additional strain used, DA464, has a mutation in the *eat-5* innexin gap junction, which shares structural and functional similarities with human connexin gap junctions, mutations of which are associated with atrial fibrillation.^47^ The response of JD21 and DA464 to commercially available cardiac drugs, PL and RE, followed the expected results (Figure S5). PL reduced the pharynx pumping rate ∼3-fold (65 pumps/min) in all three strains. RE did not significantly increase the pumping rate in wild-type but induced the maximum increase in the DA464 strain (1.5 times compared to untreated worms) and an intermediate effect (1.2 times) in JD21 worms (Figure S5). The pharynx pumping rate after Ppy NPs exposure increases in all three strains (although not statistically significant in N2 worms), with the maximum effect observed in the JD21 strain (Figure 7b), suggesting that Ppy might mainly rescue the effects of defective calcium transport.

### Treatment Duration and Excretion

**Figure 8:**
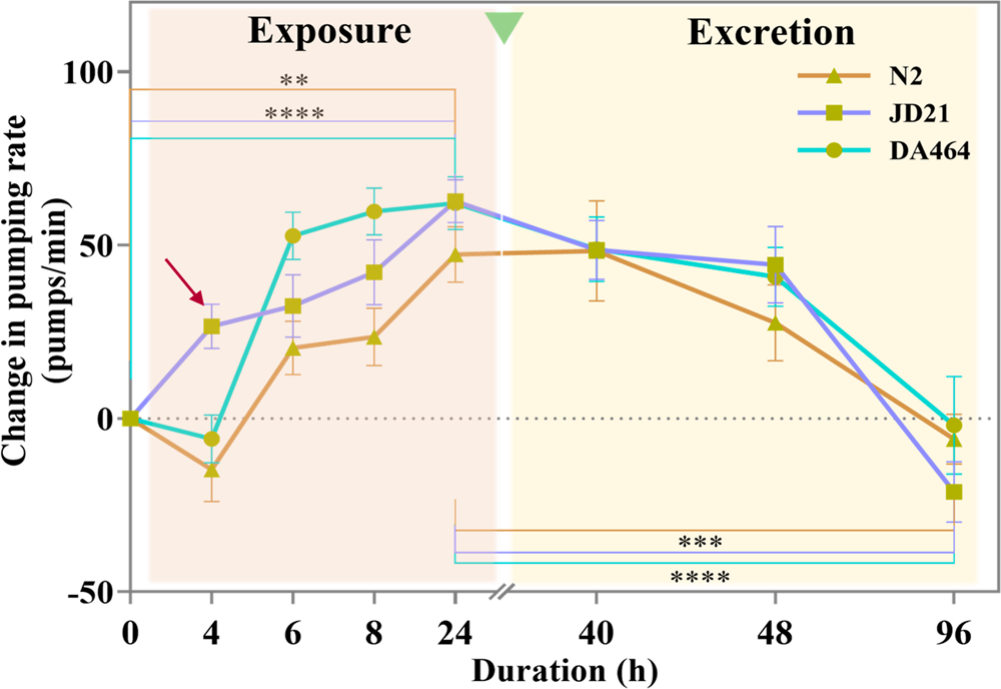
Effect of the pumping rate change during exposure and after excretion of PPy NPs over time was evaluated in N2 (wild-type), JD21 (*cca-1* calcium channel mutant), and DA464 (*eat-5* gap junction mutant) (*N* ≈ 30).

Four exposure durations were chosen, 4, 6, 8, and 24 h, and the pharynx pumping rate increased in a time-dependent manner in all three strains (Figure 8). The change in pumping rate was computed by normalizing the pumping rate of treated worms with the mean pumping rate of untreated worms. Interestingly, the effect of Ppy was noticeable early in JD21, in the first 4 h of treatment (Figure 8, arrow mark). For the DA464 strain, a notable increase in pumping rate was observed at 6 h which remained nearly constant up to 24 h. In comparison, the pumping rate of the wild-type strain increased over time and reached a maximum of 24 h, but with a lesser increase in pumping rate than the other two slow-pumping strains.

The pharynx pumping rate evaluated after the excretion of the Ppy NPs (for 16, 24, and 72 h) shows the pharyngeal effect sustained up to a day and reached the basal rate (the pumping rate of untreated worms) after 72 h of excretion (Figure 8), similar to the observed effect of PL and RE (Figure 5). Table S1 includes the change in the pumping rate at each time and the computed slope. We could see that the slow pumping strains were more affected by Ppy NPs exposure since the rate increased faster and the slope was higher, while the decrease to the basal value was similar for the three strains. We observed that the worms from both the treated and untreated groups were healthy in terms of survival and behavior during the experiment’s time, showing no apparent anomalies.

### Lipid Oxidation, Accumulation, and Metabolism

**Figure 9:**
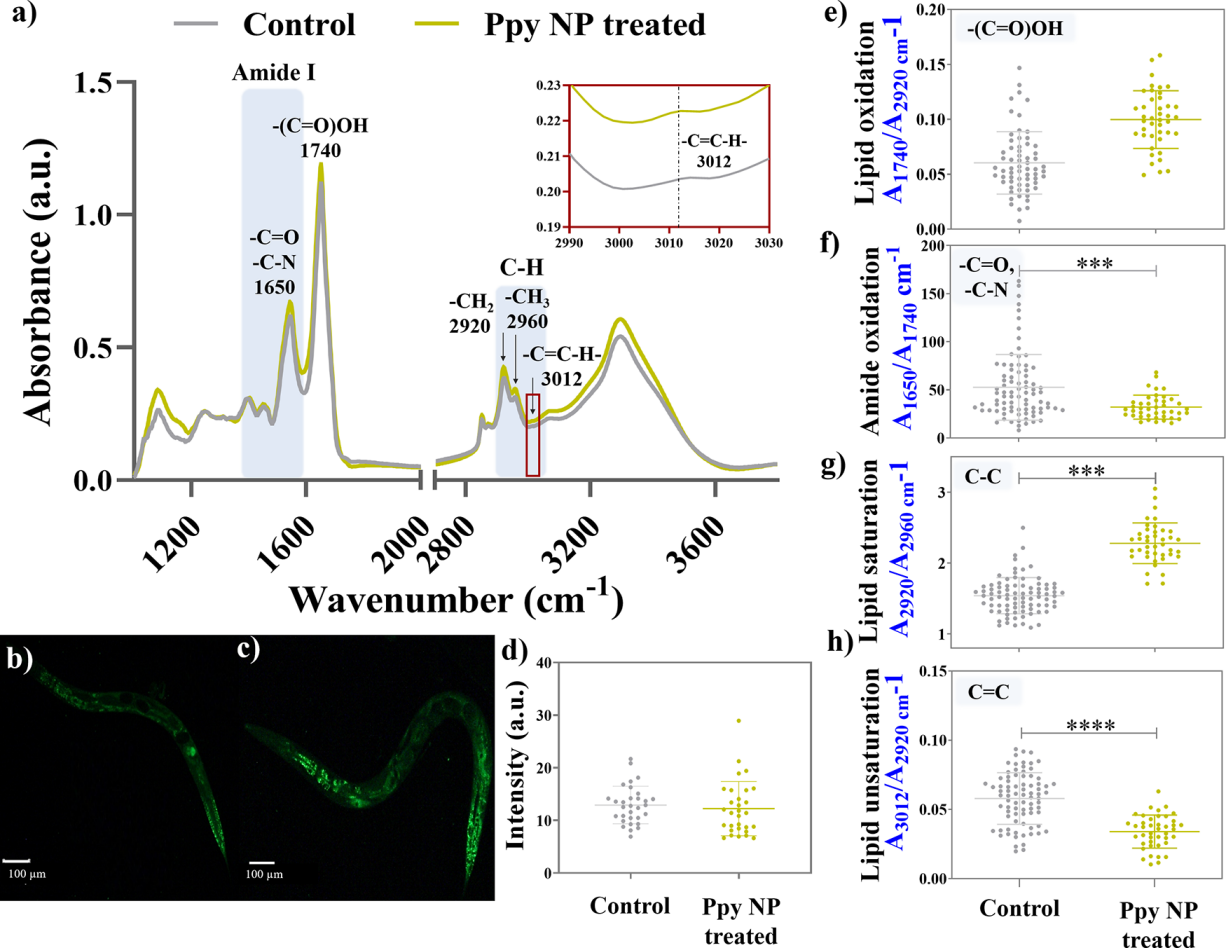
(a) FT-IR spectra of untreated and treated *C. elegans*. (b) Fluorescent images of BODIPY stained control worms. (c) Fluorescent images of BODIPY stained Ppy treated worms. (d) Quantification of the lipid levels computed using BODIPY intensities, measurements of the (e) lipid oxidation ratio (*A*_1740_/*A*_2920_), (f) Lipid saturation ratio (*A*_2920_/*A*_2960_), and (g) lipid unsaturation ratio (*A*_3012_/*A*_2920_) of untreated (control) and treated worms (Ppy NP treated) using SR-μFTIR spectroscopy (*N* ≈ 50 worms).

One of the recurrent side effect of drugs is their impact on lipid metabolism and lipid levels. Using *C. elegans* and the implementation of analytical techniques from biology to physics, we can estimate levels of specific molecules. SR-μFTIR spectra (Figure 9a) and BODIPY staining provided information about the lipid levels of *C. elegans* after exposure to Ppy NPs. The lipid staining with BODIPY indicated that lipid accumulation is unaffected upon Ppy NPs treatment (Figure 9b–d).

SR-μFTIR spectroscopy facilitates a detailed study of the lipids in *C. elegans* by assessing the lipid oxidation and metabolism upon Ppy treatment through relative absorbance ratios of functional groups. A higher amount of ester bonds from carbonyl groups (∼1740 cm^–1^) suggests enhanced lipid oxidation, estimated by the lipid oxidation ratio: *A*_1740_/*A*_2920._^48^ The lipid oxidation seems to increase slightly after treating the worms with Ppy NPs (Figure 9e), although statistically nonsignificant.

Similarly, we assessed lipid metabolism by estimating the amount of saturated lipids (−C–H_2_ groups) and unsaturated lipids (−C=C–H) through the ratios *A*_2920_/*A*_2960_ and *A*_3012_/*A*_2920_, respectively. The Ppy treatment caused an increase in saturated lipids (C–C) and a lower unsaturation (C=C) compared to untreated worms (Figure 9f,g), implying an enhanced lipid metabolism.^49^ The improved lipid metabolism is probably caused by an increased pumping rate of Ppy-treated worms, leading to a rise in energy and fuel required, which also aligned with the BODIPY staining, showing no change in total lipids. As expected, the amount of unsaturated fatty acids shows a contrasting effect compared to the saturated fatty acid content. To summarize, Ppy NPs do not cause a significant oxidative impact or lipid accumulation. An increase in lipid metabolism is observed, probably due to higher pumping, causing higher energy consumption.

### Calcium Transient Levels

Ca^2+^ plays a significant role in signal transduction in muscle contractions. Several literature studies also reported that Ppy affects calcium signaling through voltage-gated calcium channels and gap junctions.^7,14^ The changes in pharynx pumping rate, treatment duration, and post-excretion indicated that the pharyngeal effects were more substantial on the JD21 strain than N2 and DA464. Interestingly, the JD21 strain possesses a mutation in the voltage-gated calcium channel *cca-1*, suggesting that the mechanistic action of Ppy NPs could be mediated through Ca^2+^ signaling.

**Figure 10:**
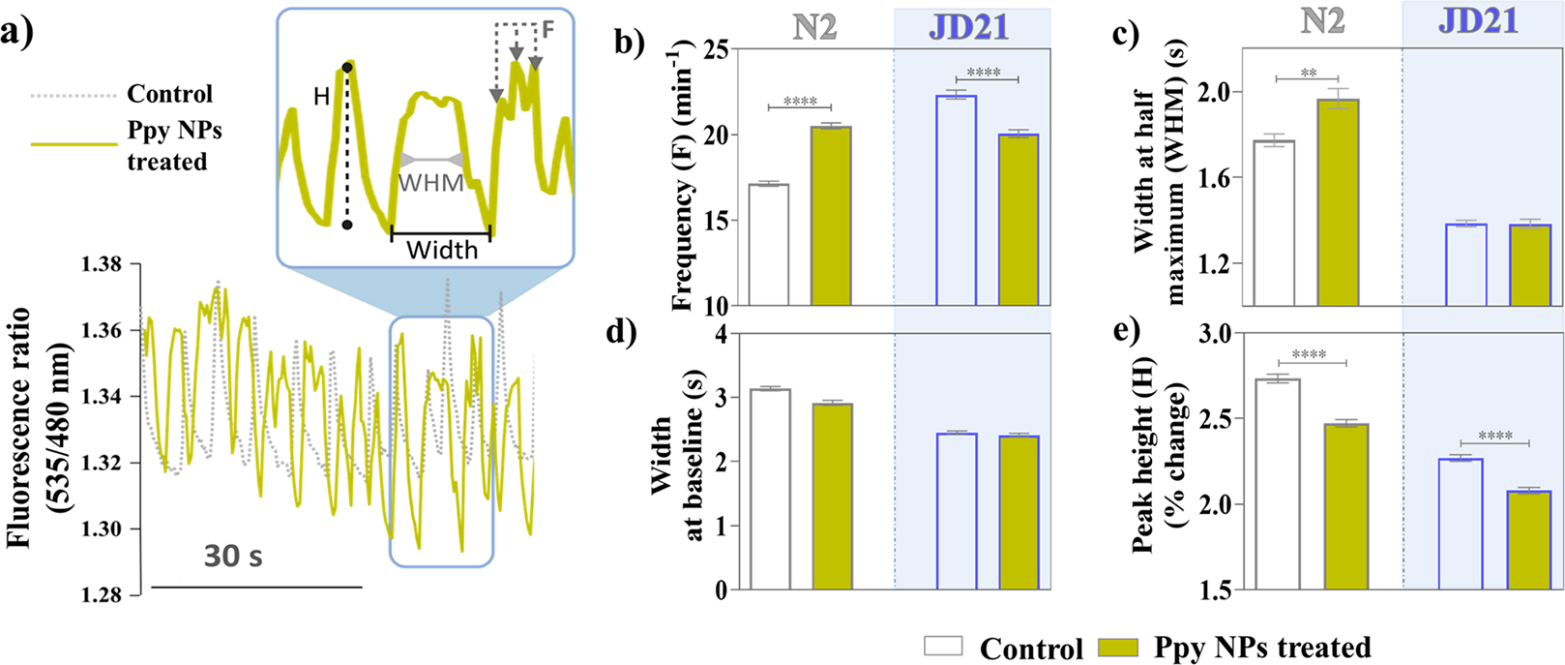
Calcium transients in the pharynx are measured by fluorescent calcium imaging. (a) Calcium transient spikes in control and Ppy NP treated worms showing the frequency (*F*), width at half-maximum (WHM), width at baseline (Width), and peak height (*H*). (b) Quantification of the frequency of calcium transients, (c) width at half-maximum, (d) width at baseline, and (e) peak height of untreated and Ppy NP treated worms in N2 and JD21 strains.

Consequently, we measured the pharyngeal Ca^2+^ transient levels in untreated and Ppy NP-treated worms of the N2 and JD21 strains using calcium imaging. In order to perform the [Ca^2+^] measurements, we utilized the worm strains AQ2038^50^ and JD21-Ca^2+^, derived from the N2 and JD21 strains, respectively, by expressing the fluorescent Ca^2+^ sensor YC-2.1 in pharyngeal muscle cells. Pharynx pumping videos and Ca^2+^ transient spikes were recorded in worms with and without NP treatment. Figure 10a illustrates a snapshot of the recordings. After pumping at a high rate for a long time, worms are energy depleted and fatigued. The increase in lipid metabolism also corroborated the increased consumption and subsequent energy depletion we observed previously by SR-μFTIR. Due to this energy depletion, more square waves are observed at this stage rather than sharp, narrow spikes, resulting in a lower spikes frequency despite a higher pumping rate. Therefore, a lower frequency and a higher peak width imply the presence of more square waves in the Ca^2+^ transient records. This pattern was observed in the case of Ppy-treated worms (Figure 10a, green), corroborating the increase in the pumping rate in NP-treated worms compared to untreated worms.

The mean frequency, width at half-maximum and baseline, and the height of the peaks were computed from 10 min recordings for 20 worms in each condition. For the N2 worms, the frequency of Ca^2+^ spikes increased upon Ppy treatment in wild-type worms (Figure 10b). Interestingly, in the presence of Ppy NPs, the width at half-maximum increased (Figure 10c), while the width at the baseline did not change (Figure 10d). In the presence of Ppy NPs, the worms display square-shaped peaks with an extended stay of Ca^2+^ at a high level before returning to the baseline (Figure 10a, green). The mechanism for this effect of the Ppy NPs is unclear but could be mediated by the direct interaction of the Ppy NPs with the pharynx muscle cells. When the Ppy NPs treatment was given to JD21 worms, a strain possessing T-type Ca^2+^ channel *cca-1* deletion mutation, the Ppy NPs did not affect the width of the peaks. The different response to Ppy NPs on wild-type and *cca-1* deleted strains suggests that the T-type Ca^2+^ channel *cca-1* plays a central role in the effect of Ppy NPs treatment. Further investigation of the precise molecular mechanism of action of Ppy NPs on pharynx pumping rate could shed light on their role in influencing the cardiac rhythm.

## Conclusions

*C. elegans* was validated as a small animal platform to test systemic and pharyngeal effects using two substances with known cardiac effects: Propranolol (PL) and Racepinephrine (RE). The commercial drugs (PL and RE) did not exhibit any adverse toxic effects in *C. elegans*, and their performance was as reported in cardiac therapies. Ppy NPs were biocompatible in *C. elegans* up to the highest concentration of 500 μg/mL measured. Other toxicity parameters, such as growth and development, reprotoxicity, intergenerational toxicity, lipid accumulation, and oxidative effects, showed no toxic effects or change upon treatment. Thus, Ppy NPs were deemed safe for use in *C. elegans.*

Ppy NPs influenced the pharyngeal pumping rate of *C. elegans*, confirming its potential impact on the cardiac rhythm. The effect on the pharynx pumping rate was time-dependent and sustained up to 24 h after withdrawal of the treatment, suggesting a moderate lasting impact. We postulate that the possible mechanism of action of Ppy NPs could be through calcium signaling. Treatment with Ppy NPs impacted the frequency of calcium oscillations, indicating a likely increase in calcium levels, which was evaluated in the strains with fluorescent pharynx, AQ2038, and JD21-Ca^2+^. The difference in the effect between wild-type worms and the voltage-gated T-type calcium channel (*cca-1*) deleted strain suggested an essential role of voltage-gated calcium channels in the mechanistic action of Ppy NPs.

*C. elegans* shows tremendous potential as a small animal model for arrhythmia research, although limitations should be kept in mind. These 3R models can be used for an initial screening to obtain safe-by-design nanomaterials, but rigorous evaluation with larger animals is obligatory to progress to clinical trials.

## Experimental Section

### Materials

All reagents were bought from Sigma-Aldrich unless stated otherwise. Pyrrole monomer (Py), Poly(vinyl alcohol) (PVA), and ferric chloride hexahydrate (FeCl_3_·6H_2_O) were used for Ppy NP synthesis. Propranolol (PL) (AstraZeneca) and racepinephrine (RE) are the commercial compounds tested. Agar and peptone (CondaLab), sodium chloride, magnesium sulfate, cholesterol, and calcium chloride were the components of the nematode growth medium (NGM). For the growth and culture of OP50 *E. coli* bacteria, broth media was prepared using tryptone, yeast extract (CondaLab), sodium chloride, and sodium hydroxide were utilized. We used 10 mM sodium azide for imaging the worms and 2% agarose to prepare agar pads for recording videos of the alive worms under the microscope.

### Synthesis and Characterization of Ppy NPs

Polypyrrole nanoparticles (Ppy NPs) were synthesized using a chemical oxidative polymerization method following a previously published protocol.^35^ We characterized the synthesized Ppy NPs through DLS, scanning electron microscopy (SEM), TEM, FT-IR, and UV–vis-NIR to confirm their physicochemical and structural properties. The stability of Ppy NPs in NaOH, household bleach (sodium hypochlorite), and M9 buffer used in *C. elegans* experiments was evaluated by DLS at *t* = 0 and *t* = 20 min.

### *C. elegans* Growth and Maintenance

The *C. elegans* strains (N2, JD21, and DA464) and the *E. coli* strain OP50 used as the bacterial food source was obtained from Caenorhabditis Genetic Center (CGC) stock collection, University of Minnesota, St. Paul, MN, USA. The wild-type (N2) and two slow-pumping strains (JD21 and DA464) were cultured and maintained in NGM agar plates containing OP50 at 20 °C in an incubator. Experiments were performed with a synchronized population obtained using a standard bleaching protocol.^51^ Synchronized worms arrested at the L1 stage after bleaching were transferred to fresh OP50 containing NGM plates and allowed to grow until the L4-young adult (L4-YA) stage (∼42–45h) for treatments with PL, RE, and Ppy NPs.

### *C. elegans* Exposure to Ppy NPs, PL, and RE

We used M9 buffer as the aqueous solution for exposure of worms to Ppy NPs, PL, and RE. Worms synchronized at the L4-YA stage were collected with M9 buffer and cleaned thrice by centrifugation at 4400 rpm for 1 min to wash off excess OP50. Then, the worms were exposed to the three substances in a liquid exposure system in 96-well plates at 20 °C. Heat-inactivated OP50 with an optical density of 1.0 (at 600 nm) was used as the food source during the exposure. The bacteria were incubated at 85 °C for 30 min, centrifuged for 5 min at 6,000 rpm, and redispersed in M9 buffer. To each well, we added 50 μL M9 buffer consisting of 3 μL OP50 and ∼25–30 worms and 50 μL of the NPs solution or the drugs dispersed in MQ water, so that total volume per well is 100 μL. Unless otherwise stated, the standard exposure duration is 24 h, and the concentration is 100 μM for PL and RE and 100 μg/mL for Ppy NPs.

### Recovery of Ingested NPs

After the worms were treated with Ppy NPs, ingested NPs could be recovered through bleaching. The control and NP-treated worms were washed with M9 buffer 3–4 times to remove excess NPs and debris entirely from the solution. 25 μL of 5 M NaOH and household bleach and 875 μL of M9 were added to ∼100 μL of worm pellet (or NP solution), followed by vortexing for 2 min. The process is repeated twice. The solutions were continuously monitored for the worms’ complete dissolution, after which the bleaching was stopped by adding 0.5 mL of M9 buffer. We washed the solutions twice with M9 buffer and visualized them under an optical microscope before TEM and DLS analysis.

### Imaging and Compatibility Assays

The survival rate of *C. elegans* upon exposure to Ppy NPs was computed in three NP concentrations: 20, 100, and 500 μg/mL, and the survival rate after PL and RE treatments was measured for 100 μM concentration. After 24 h of aqueous exposure, the number of alive-to-dead worms was counted in each treatment group, and the survival rate was calculated. However, the highest Ppy NP concentration, 500 μg/mL, caused experimental difficulties in cleaning and visualization. Therefore, the subsequent assays were performed with the intermediate concentration, 100 μg/mL. The worms were washed by centrifugation to remove excess NPs or drugs, OP50, and other impurities until a clear supernatant was obtained. The worms are pelleted down to acquire a concentrated solution (about 50 μL) containing *C*. *elegans*. A drop of the worms is placed on a glass slide. An equal volume of 10 mM sodium azide is added to immobilize the worms and sealed with a coverslip for microscopic imaging. Body length is measured using ImageJ from the images obtained.

**Figure 11:**
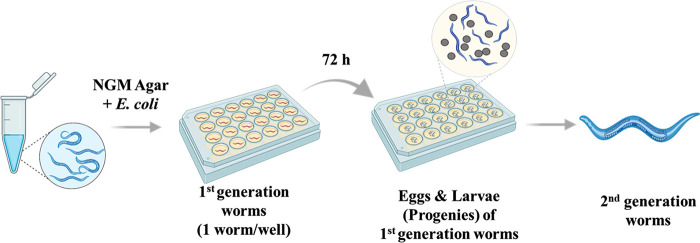
Reproductive ability and intergenerational toxicity assays of *C. elegans* after exposure. Created with BioRender.com.

After cleaning, 9 worms from each treatment group were individually placed on an NGM-agar plate, and the number of progeny for 72 h was computed to estimate reproductive toxicity. Likewise, ∼30 eggs laid by the treated worms from each treatment group were picked, transferred to a new plate, and monitored for 72 h. Body length was estimated for the progenies to investigate intergenerational growth and developmental toxicity (Figure 11).

### *C. elegans* Pharynx Pumping

**Figure 12:**
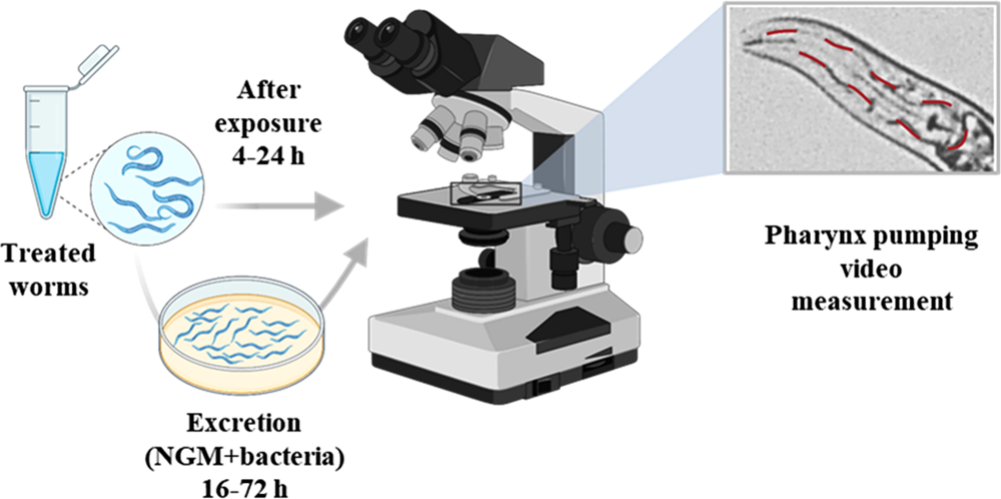
Experimental process of pharynx pumping rate measurements after exposure and excretion. Created with BioRender.com.

The protocol to measure pharynx pumping is as mentioned in the Wormbook Methods.^34^ Briefly, agar pads are prepared using 2% agarose on a glass slide to visualize the *C. elegans*’ pharynx for counting the pumping rate. A small drop of about 5 μL of OP50 is placed on the agar pad and dried to ensure feeding and pumping during counting. Approximately 10–15 worms are placed in each agar pad over the OP50 colony and left to dry. Around 7–10 min of incubation time is necessary for the worms to acclimatize to the new environment and start moving. A coverslip is placed on top to ensure the worms did not move too fast and hinder the counting. The slides were then visualized under the microscope at 100× magnification, 30 s clips of each worm were recorded, and the pumping rate was counted. 10–15 worms in each control and treated group were measured during each experiment, and the experiment was repeated 3 times, amounting to ∼30 worms per treatment condition. The change in pharynx pumping rate was also computed after 4, 6, 8, and 24 h to assess the effect of treatment durations on the pharyngeal pumping. Similarly, after exposure and cleaning, the worms were placed on NGM agar plates containing OP50 bacteria to allow for bacterial feeding that facilitates the excretion of the ingested substances. The pharynx pumping rate was measured 16, 24, and 72 h after excretion to understand whether the pharyngeal effects can be sustained post-excretion (Figure 12).

### Lipid Staining of *C. elegans*

Intracellular lipid levels in treated and untreated worms were quantified by using fluorescent staining with BODIPY as the fluorescent dye. After 24 h of exposure, worms are washed in M9 buffer to clean them from debris and excess uningested drugs or NPs. After the final wash, the supernatant is discarded, leaving a small volume (∼100 μL) of worm pellet, and 1 mL of BODIPY (35 μM) is added to the worm pellet and incubated under shaking for 30 min. The worms are thoroughly washed with M9 buffer after 30 min to ensure the excess dye is removed. A drop of worms is placed on a glass slide, and 10 mM tetramisole dispersed in water is added to it to paralyze the worms before imaging. The fluorescence intensity from the images obtained was then quantified using ImageJ and compared between the different treatment groups.

### Synchrotron Fourier Transform Infrared Spectroscopy

We employed synchrotron-radiation-sourced FT-IR spectroscopy (SR-μFTIR) to characterize the biochemical compositions of the untreated and treated worms. Worms at the fully grown adult stage are more difficult to penetrate by the incident rays. Therefore, to have a better intensity of the FTIR spectra, ∼500 L3 stage worms are treated with the substances in Milli-Q water for 16 h, washed by centrifugation, transferred dropwise onto a 9 mm CaF_2_ window (∼45 worms per window), and vacuum-dried. The SR-μFTIR analysis was conducted at the MIRAS beamline at ALBA synchrotron, Spain using a Hyperion 3000 Microscope coupled to a Vertex 80 spectrometer (Bruker) equipped with 36× magnification objective from 900–4000 cm^–1^. The spectra were collected in transmission mode using an MCT detector at 4 cm^–1^ spectral resolution and 8 × 8 μm^2^ aperture dimensions. Background signal was scanned in each CaF_2_ window from an area free of worms. FTIR spectra were extracted using OPUS 7.5 (Bruker), noise removal, baseline correction, and application of Savitsky–Golay second derivative were performed using Unscrambler and Origin 2019b. Relative absorbance ratios were calculated from the second derivative obtained from the FTIR spectral data. Four ratios, namely, lipid oxidation (A1740/A2920), saturation (A2920/A2960), and unsaturation (A3012/A2920) levels were evaluated.

### Calcium Transient Levels

For the Ca^2+^ imaging experiments, we used AQ2038 as the wild-type strain (N2 strain expressing the YC2.1 fluorescent Ca^2+^ sensor in pharyngeal muscle cells, kindly provided by Drs. Robyn Branicky and W. Schafer, M.R.C. Laboratory of Molecular Biology, Cambridge, United Kingdom). We crossed the JD21 and AQ2038 strains to produce JD21 expressing the Ca^2+^ sensor in pharyngeal muscle cells (JD21-Ca^2+^). For the crosses, AQ2038 males were crossed with JD21 hermaphrodites, and the adult F1 generation worms were picked on day 3. Fluorescent males from the F1 progeny crossed again with JD21 hermaphrodites until fluorescent mutant hermaphrodites were achieved. The resulting genetically modified worms were genotyped through PCR to ensure the crosses were successful and the fluorescent probe had been incorporated. The resulting worms were treated with Ppy NPs at 100 μg/mL for 24 h at 20 °C in a 96-well plate, in the same manner as they were treated for the remaining assays. After exposure, the worms were cleaned using M9 to remove excess bacteria and debris and transferred to NGM plates. The worms are then picked from the plates, placed on 1% agar pads containing bacteria, and immobilized in the measurement chamber using glue (Dermabond Topical Skin Adhesive, Johnson & Johnson, New Brunswick, NJ, USA). The videos of pharyngeal pumping motion and corresponding Ca^2+^ peaks were recorded for 10 min for each worm, using a Zeiss Axiovert 200 inverted microscope. Fluorescence records were analyzed using the Metafluor program (Universal Imaging). The traces shown were obtained as the ratio of the simultaneous images acquired at 535 nm emission and 480 nm emission (under 430 nm continuous excitation). Fluorescence data were then analyzed with a specific algorithm designed to calculate the width, intensity, and frequency of the Ca^2+^ peaks in each experiment.

### Statistical Analysis

Graphical representation of all the data and their statistical analysis were made in GraphPad Prism 9.4. Data comparing two treatment groups were analyzed using Mann–Whitney tests, and Kruskal–Wallis was used to compare multiple groups. A *P* > 0.05 is considered nonsignificant, and no statistics are displayed in the graphs where no statistical significance was observed between treatment groups, and the significance levels with their corresponding *p*-values are * = *P* ≤ 0.05, ** = *P* ≤ 0.01, *** = *P* ≤ 0.001, **** = *P* ≤ 0.0001, for data represented with a significance.

## Supporting Information Available

Synthesis of Ppy NPs; characterization by DLS, SEM, TEM, FT-IR, UV–vis-NIR, and TGA; optical microscopic image and hydrodynamic diameter of ingested NPs; survival rate of worms after treatment with PL and RE; pharynx pumping rate of *C. elegans* upon treatment with Ppy NPs; rate of increase between 0 and 24 h; and rate of decrease between 24 and 96 h for N2, JD21, and DA464 (PDF).
