# Supplementary material for: Arrhythmic Effects Evaluated on Caenorhabditis elegans: The Case of Polypyrrole Nanoparticles
Source: ACS Nano. 2023 Aug 25;17(17):17273–84. doi: 10.1021/acsnano.3c05245 (PMC10510705; doi:10.1021/acsnano.3c05245)
Supplement: Supplementary file 1 — nn3c05245_si_001.pdf [file nn3c05245_si_001.pdf]

SUPPLEMENTARY INFORMATION

Arrhythmic Effects Evaluated on *Caenorhabditis elegans:* The Case of Polypyrrole Nanoparticles

Sumithra Yasaswini Srinivasan[1], Pilar Alvarez Illera[2], Dmytro Kukhtar[1,3], Nuria Benseny-Cases[4#], Julián Cerón[2], Javier Álvarez[2], Rosalba I Fonteriz[2], Mayte Montero[2], Anna Laromaine[1]*

1. Universitat de Autonoma de Barcelona, Institut de Ciència de Materials de Barcelona (ICMAB), 08193, Bellaterra, Barcelona, Spain

2. Universidad de Valladolid, Instituto de Biomedicina y Genética Molecular (IBGM), 47005, Valladolid, Spain

3. Modeling Human Diseases in *C. elegans* Group - Genes, Disease and Therapy Program, Bellvitge Biomedical Research Institute - IDIBELL, 08908 L'Hospitalet de Llobregat, Barcelona, Spain.

4. ALBA Synchrotron, 08290, Cerdanyola del Vallès, Barcelona, Spain.

Present Addresses

Universitat de Autònoma de Barcelona, Unitat de Biofísica, Departament de Bioquímica i Biologia Molecular, 08193 Bellaterra, Spain.

Synthesis of Ppy NPs

The Polypyrrole Nanoparticles (Ppy NPs) were synthesized by chemical oxidative polymerization method (Figure S1). Briefly, pyrrole (0.1 M), being the monomer of interest, was added to a solution of $FeCl_3.6H_2O$ oxidant (24:1 to monomer ratio) and PVA surfactant (7.5 wt% of monomer). The mixture was sonicated at 5 ˚C for 4 hours to complete the polymerization reaction and washed thrice with distilled water until a clear supernatant was observed. The pellet was redispersed in distilled water and maintained at 4 ˚C for future use.

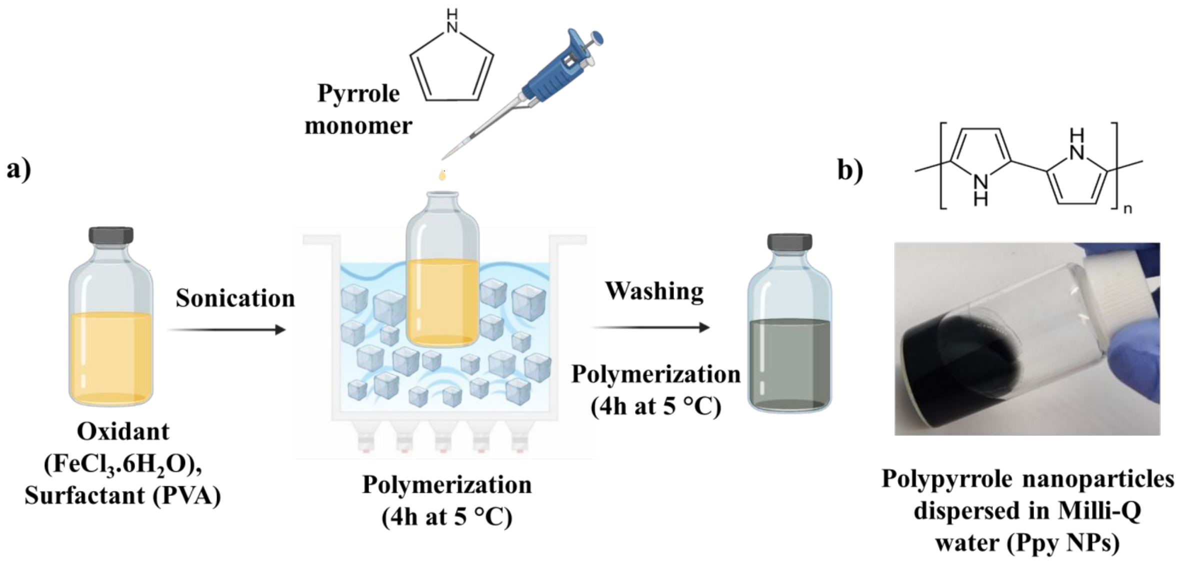

Figure S1. a) Synthesis of Ppy NPs by oxidative polymerization b) Ppy NPs uniformly dispersed in water

Characterization of Ppy NPs

The as-synthesized Ppy NPs were dried at 60 ˚C to obtain dry powders for physicochemical characterization. The size and morphology of the NPs were examined by electron microscopy

and dynamic light scattering (DLS) techniques. For the scanning electron microscopy (SEM) and transmission electron microscopy (TEM) analysis, aqueous dispersion of Ppy NPs were prepared at 10 µg/mL concentration, and the mean diameter was 132±31 nm. The SEM and TEM images revealed that the NPs exhibits a uniform spherical morphology (Figure S2a, b). Likewise, for the DLS analysis, we prepared 100 µg/mL dispersion in Milli-Q water and the scan was performed with 10 runs/measurement. The hydrodynamic diameter of Ppy NPs as revealed by DLS was 196±62 nm with a polydispersity index of 0.17 (Figure S2c). The PDI corroborates well with the dispersion stability of the NPs solution (Figure S1b). The NPs´ sizes were reproducible and agreeing with the previous reports using this protocol.

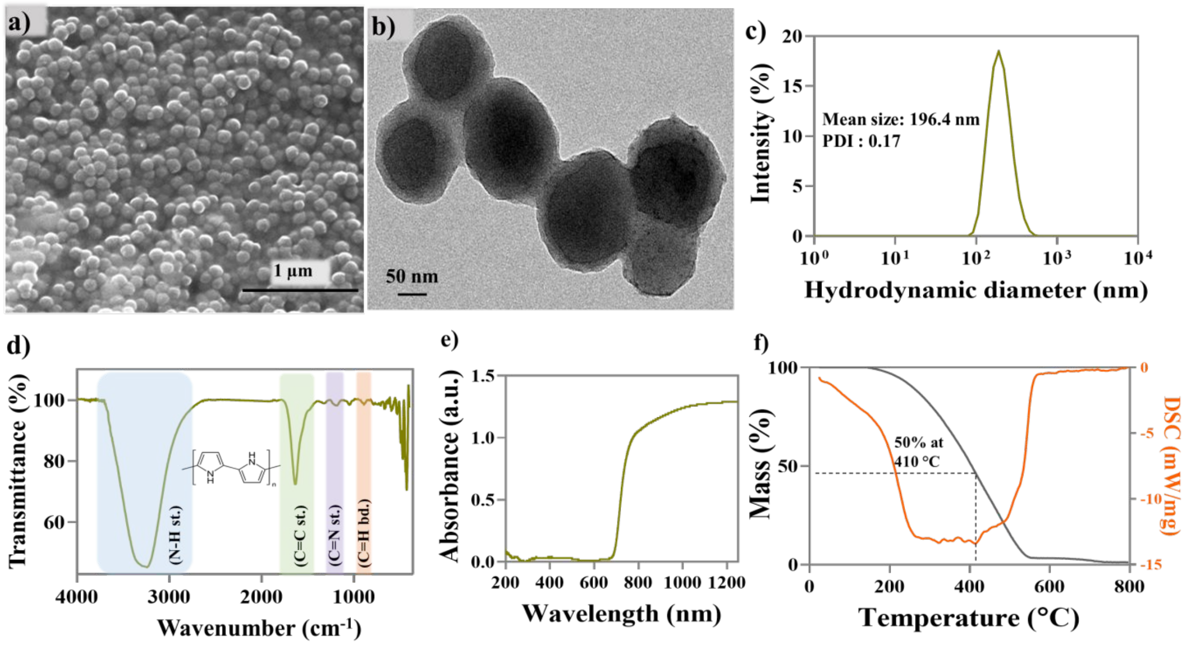

Figure S2. Size and Morphology of Ppy NPs a) SEM image, b) TEM image, c) hydrodynamic diameter of Ppy NPs, d) FTIR spectra elucidating the chemical structure, e) UV-Vis-NIR

spectra showing its optical absorbance, and f) TGA spectra of Ppy NPs confirming its thermal stability.

The chemical structure of Ppy NPs can be appreciated from the FTIR spectra (Figure S2d). The broad intense peak at 3311 cm^{-1} is due to N-H stretching of secondary amines and the peak at 1631 cm^{-1} occurs from C=C stretching of conjugated alkenes. The smaller peaks at 1324 and 862 cm^{-1} correspond to C-N stretching from aromatic amines and C-H bending in alkane chains, respectively. Similarly, the Ppy formation was also confirmed by UV-Vis-NIR spectra, where the NPs displayed a typical broad and intense absorbance in the NIR region (700-1200 nm). The thermal degradation behavior of Ppy NPs (≈ 1 mg) was studied by thermo-gravimetry analysis. The NPs were heated up to 800 ˚C, with a heating rate of 10 ˚C/min. The TGA spectra shows that Ppy NPs possess exceptional thermal stability, with a gradual decomposition rate and maximum degradation between 200-400 ˚C.

Recovery of Ingested NPs

After employing the bleaching protocol to digest worms and recover the ingested NPs, the sample is cleaned thoroughly with Milli-Q water and immediately visualized in optical microscopy (Figure S3) prior to TEM analysis, to ensure that the worms are digested, but the NPs are present. The hydrodynamic diameter and polydispersity index are also measured to

study the aggregation behaviour of the ingested NPs and found to be 311±75 nm and 0.3, respectively.

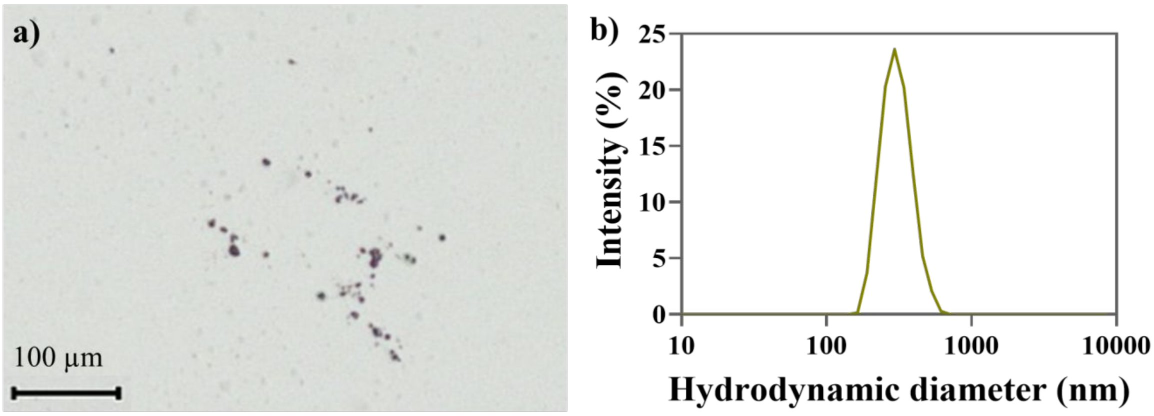

Figure S3. The ingested Ppy NPs recovered from *C. elegans* a) visualized by optical microscopy and b) hydrodynamic diameter of the recovered nanoparticles.

Survival rate

The worms exposed to 100 µM of PL and RE for 24 hours were scored for survival after exposure, to estimate whether PL and RE are compatible in *C. elegans*. Both the substances showed ≈99% survival, validating the suitability to be analysed in *C. elegans.*

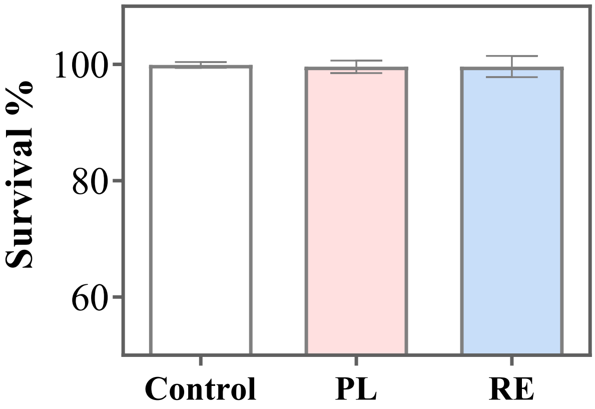

Figure S4. The survival rate of *C. elegans* after 24 hours treatment with PL and RE at 100 μM concentration.

Pharynx pumping rate

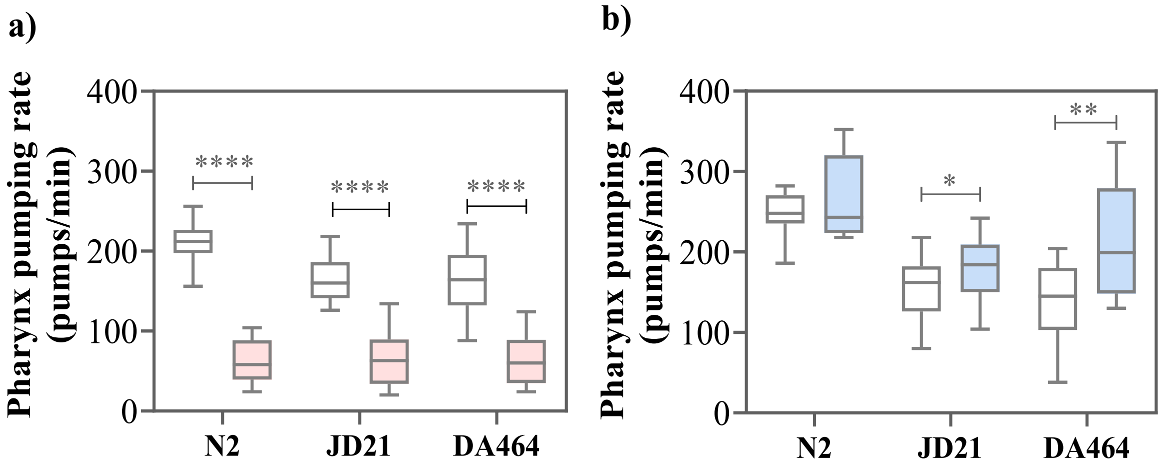

Figure S5. a) Pharynx pumping rate of PL treated and b) RE treated N2, JD21, and DA464 worms (N≈30), compared with the respective untreated worms.

Table S1. The mean change in pumping rate (pumps/min) of Ppy NPs treated N2, JD21, and DA464 *C. elegans*, the rate of increase from 0 to 24 hours, and rate of decrease from 24 to 96 hours.

Strains	Change in Pumping rate (Pumps/min)							Rate of increase (pumps/h)	Rate of decrease (pumps/h)
Duration (h)	4	6	8	24	40	48	96	0-24h	24-96h
N2	-20±10	20±8	24±8	47±8	48±14	28±11	-6±7	1.3	0.8
JD21	27±6	31±9	42±9	63±6	49±9	44±11	-21±9	2.0	1.2
DA464	-6±7	53±7	60±7	62±8	49±9	41±8	-2±14	2.0	0.9
